# Effects of Dry Needling of Latent Trigger Points on Viscoelastic and Muscular Contractile Properties: Preliminary Results of a Randomized Within-Participant Clinical Trial

**DOI:** 10.3390/jcm10173848

**Published:** 2021-08-27

**Authors:** Albert Pérez-Bellmunt, Oriol Casasayas-Cos, Carlos López-de-Celis, Jacobo Rodríguez-Sanz, Jorge Rodríguez-Jiménez, Sara Ortiz-Miguel, Toni Meca-Rivera, César Fernández-de-las-Peñas

**Affiliations:** 1 Basic Sciences Department, Universitat Internacional de Catalunya, 08195 Sant Cugat del Vallès, Spain; aperez@uic.cat (A.P.-B.); casasayascos@uic.es (O.C.-C.); jrodriguezs@uic.es (J.R.-S.); sortiz@uic.es (S.O.-M.); tonimeca@uic.es (T.M.-R.); 2 ACTIUM Functional Anatomy Group, 08017 Barcelona, Spain; 3 Fundació Institut Universitari per a la Recerca a l’Atenció Primària de Salut Jordi Gol i Gurina (IDIAPJGol), 08007 Barcelona, Spain; 4 Department of Physical Therapy, Occupational Therapy, Physical Medicine and Rehabilitation, Universidad Rey Juan Carlos, 28922 Alcorcón, Spain; jorge.rodriguez@urjc.es; 5 Cátedra de Investigación y Docencia en Fisioterapia: Terapia Manual y Punción Seca, Universidad Rey Juan Carlos, 28922 Alcorcón, Spain

**Keywords:** dry needling, myofascial pain, trigger points, gastrocnemius

## Abstract

This study aimed to evaluate changes in neuromuscular function and pain perception in latent trigger points (TrPs) in the gastrocnemius muscle after a single session of dry needling. A randomized within-participant clinical trial was conducted. Fifty volunteers with latent TrPs in the gastrocnemius muscles were explored. Each extremity was randomly assigned to a control or experimental (dry needling) group. Viscoelastic parameters and contractile properties were analyzed by tensiomyography. Ankle dorsiflexion range of motion was assessed with the lunge test. Pressure pain thresholds (PPT) and pain perceived were also analyzed. The results observed that three viscoelastic proprieties (myotonometry) showed significant differences in favor of the experimental extremity in the lateral gastrocnemius: stiffness (*p* = 0.02), relaxation (*p* = 0.045), and creep (*p* = 0.03), but not in the medial gastrocnemius. No changes in tensiomyography outcomes were found. The control extremity showed a higher increase in PPTs (i.e., decrease in pressure pain sensitivity) than the experimental extremity (*p* = 0.03). No significant effects for range of motion or strength were observed. In general, gender did not influence the effects of dry needling over latent TrPs in the gastrocnemius muscle. In conclusion, a single session of dry needling was able to change some parameters of neuromuscular function, such as muscle tone, relaxation, pressure pain sensitivity, and creep in the lateral (but not medial) gastrocnemius but did not improve strength or range of motion.

## 1. Introduction

Trigger points (TrPs) are a common musculoskeletal source of local and referred muscle pain and are classified as active or latent, depending on their relationship with symptoms [1]. The clinical difference between active and latent TrPs is that active TrPs reproduce the pain symptoms experienced by an individual [2], while latent TrPs can be present without spontaneous symptoms, and when elicited, they do not reproduce the symptoms of an individual [1]. It has been observed that latent TrPs can induce motor dysfunctions, such as stiffness, restriction of range of motion, and muscle fatigue, supporting their clinical relevance [3].

Latent TrPs in the gastrocnemius muscles are common [4,5], and their presence may increase the risk of injury in the lower extremity, particularly in individuals with a moderate level of activity and genetic predisposition [6]. In fact, repetitive micro-traumatic events, due to biomechanical alterations during gait, running, or sport practice, could lead to the development of TrPs in the lower extremity muscles [7]. Accordingly, proper evaluation and treatment of latent TrPs could be essential for the prevention of muscle injuries. In such a scenario, a small number of trials have shown that the treatment of gastrocnemius muscle TrPs reduces pain, improves health-related quality of life, and increases range of motion [8]. However, few studies have assessed the changes in neuromuscular function by using myotonometry after the treatment of latent TrPs in the gastrocnemius muscle [9,10].

Neuromuscular function is a combination of mechanical properties of the muscle that are related to basic properties, such as strength, contractibility, or flexibility. Tools used to evaluate the neuromuscular function include myometry and tensiomyography. Myometry provides viscoelastic information of the muscle throughout parameters, such as tone, stiffness, relaxation, or elasticity [11,12]. Tensiomyography assesses contractile parameters of the muscle [13,14], being the most frequent maximum radial displacement (Dm) and contraction speed (Tc). Despite the fact that myometry and tensiomyography assess muscle stiffness, it has been found that they do not analyze same properties, since they have low correlations between them [15].

A potential effect of manual therapy and dry needling approaches on stiffness of gastrocnemius muscle after the treatment of latent TrPs has been recently published [9,10]. However, no study has assessed whether dry needling of gastrocnemius muscle latent TrPs is able to generate changes in neuromuscular function through tensiomyography, myometry, pain sensitivity, and range of motion.

The objective of this randomized, within-participant clinical trial was to evaluate the effects of a single session of dry needling on latent TrPs in the gastrocnemius muscle on neuromuscular function, sensitivity to pressure pain, range of motion, and strength. The hypothesis was that a single session of dry needling into gastrocnemius muscle latent TrPs improves neuromuscular function, range of motion, and strength, and conversely decreases pressure pain sensitivity.

## 2. Materials and Methods

### 2.1. Study Design

A single-blind, within-participant clinical trial was performed. The randomization of the lower extremity, on which the needling intervention was conducted, was done by using a computer program (www.random.org, accessed on 20 May 2021). This study was approved by the local committee (CBAS201802) and conducted, according to the Declaration of Helsinki. The study was prospectively registered at clinicaltrials.gov (NCT04851743). Participants were previously informed of the procedure and declared their willingness to participate in the current trial through the signing of a written informed consent.

### 2.2. Participants

A total of 50 volunteers (100 lower extremities) were recruited from 10 May to 1 July 2021 from the general population. They were excluded if they had previous surgery or injury in the lower extremities, any underlying medical condition, pregnancy, the presence of muscle pain after strenuous exercise, or belonephobia. Participants were screened for the presence of latent TrPs in both gastrocnemius muscles, according to the following criteria: 1, palpable taut band; 2, hypersensitive spot on the taut band; and 3, pain referral to palpation which does not reproduce any familiar symptom [2].

### 2.3. Primary Outcomes: Neuromuscular Function

Viscoelastic properties and muscle contractile properties were considered primary outcomes, whereas pressure pain sensitivity, ankle dorsiflexion, and strength were considered secondary outcomes. All outcomes were evaluated before and after 2 min of intervention.

**Figure 1:**
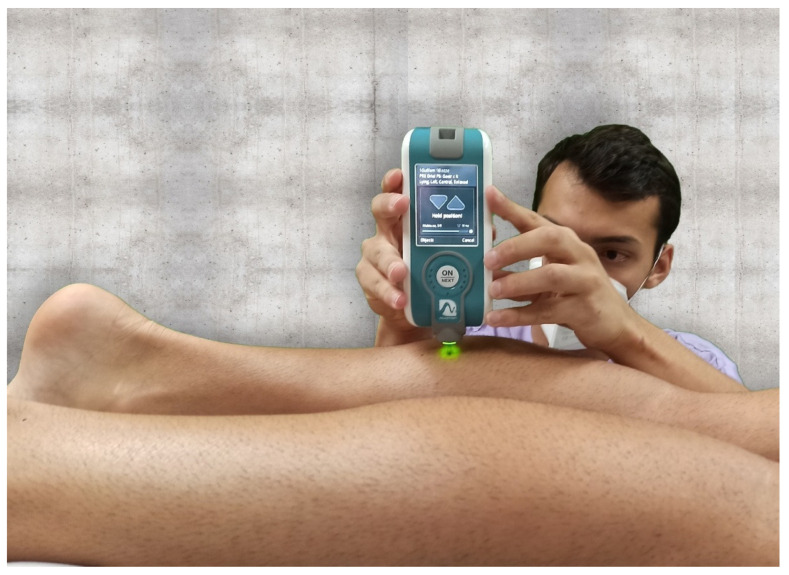
Viscoelastic properties of gastrocnemius muscles were assessed by myotonometry.

Viscoelastic properties: Viscoelastic properties of neuromuscular function were assessed with a MyotonPro instrument (MytonPro, Myoton Ltd.s., Tallin, Estonia) with participants in a prone position with the ankle slightly flexed. The MyotonPro was placed perpendicular to the skin over the muscle belly of the medial and lateral gastrocnemius (Figure 1).

The measuring device was stable while it automatically performed the predefined three trials of measurements. On each muscle, three measurements were made with an interval of 1 s between each, and the mean was used in the analysis. The reliability of the MyotonPro device has been shown to be good–excellent (ICC 0.8–0.93) in people with and without pain [9,16,17,18,19]. The oscillations recorded by the device were used to calculate the frequency that represents muscle tone. In the current study, the following variables were extracted [9,20]:

- Muscle tone (Hz): the frequency of oscillation that characterizes whether a muscle is in its passive or resting state, without any voluntary contraction.
- Stiffness (N/m): biomechanical property of a muscle that characterizes its resistance to a contraction or to the external force necessary to deform its initial shape.
- Elasticity (arbitrary units): the biomechanical property of a muscle characterizing its ability to regain its initial shape after eliminating the external force that has deformed it.
- Relaxation (ms): time taken by the muscle to regain its initial shape after deformation, following the elimination of an external force.

Muscle contractile properties: The contractile properties of neuromuscular function were obtained by using tensiomyography (TMG), again with participants in prone, with the ankle slightly flexed. All TMG parameters depend on the maximum radial displacement (Dm), which is the radial movement of the muscle belly after the application of an electrical stimulus (mm). Other parameters registered with TMG were: 1, the contraction time (Tc), which is the time between 10% and 90% of Dm [21]; 2, the delay time (Td), i.e., the time that the muscle takes to reach 10% of Dm from the start of the stimulus; 3, the relaxation time (Tr), the time between 90% and 50% of the relaxation; and 4, sustained time (Ts), the time from 50% of the displacement reached in the contraction phase to 50% of the displacement reached in the relaxation phase.

**Figure 2:**
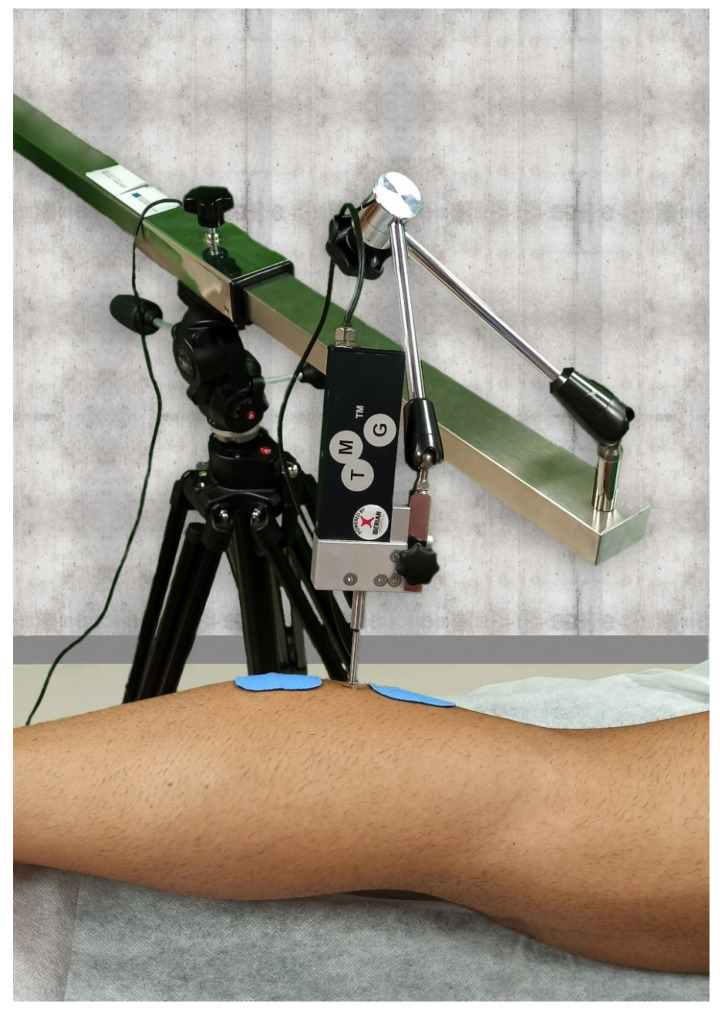
Contractile properties of gastrocnemius muscles were assessed by tensiomyography.

The measurement was performed perpendicular to the tissue with two adhesive electrodes (TMGTMG-BMCd.oo, Ljubljana, Slovenia) equidistantly placed, proximal (anode) and distal (cathode) to the sensor, with a distance of 5 cm between electrodes (Figure 2) [15]. The electrical stimulation was applied by using a TMG-100 System electrostimulator (TMGBMCd. O.o., Ljubljana, Slovenia). The amplitude was progressively increased from 20 to 100 mA, in 20 mA increments, until there was no further increase in Dm, or the maximum stimulator output was reached (i.e., 110 mA) [16]. A 10 sec-rest period was provided between stimuli to minimize the effects of fatigue and the enhancement of muscle activity [15].

### 2.4. Secondary Outcomes: Pain Sensitivity, Range of Motion, and Strength

**Figure 3:**
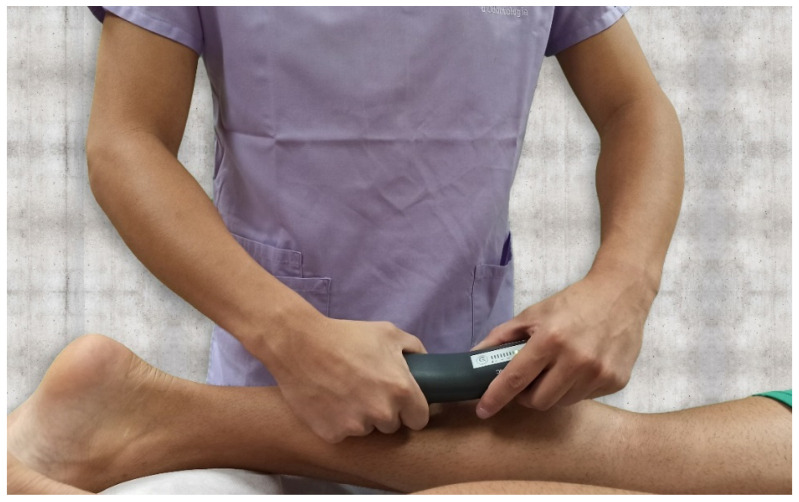
Pressure pain threshold in the gastrocnemius muscle was evaluated with a manual algometer.

Pressure pain threshold (PPT) is the minimum amount of pressure necessary to first produce a pain sensation. Pressure pain sensitivity, over the latent TrP identified in the gastrocnemius muscle, was evaluated with a manual algometer (Trigger Plus, Palpatronic, Hagen, Germany). Pressure was applied at approximately 10 N/cm^2^/s at each point until the participant reported that the pressure became painful (Figure 3). The intra-examiner reliability of this tool has been shown to be good [9,22,23]. At the time the participant perceived the pressure as painful, the evaluator asked the patient how much pain they experienced on an 11-point, numerical pain rating scale (NPRS) (0 = no pain, 10 = maximum pain) [9,24].

**Figure 4:**
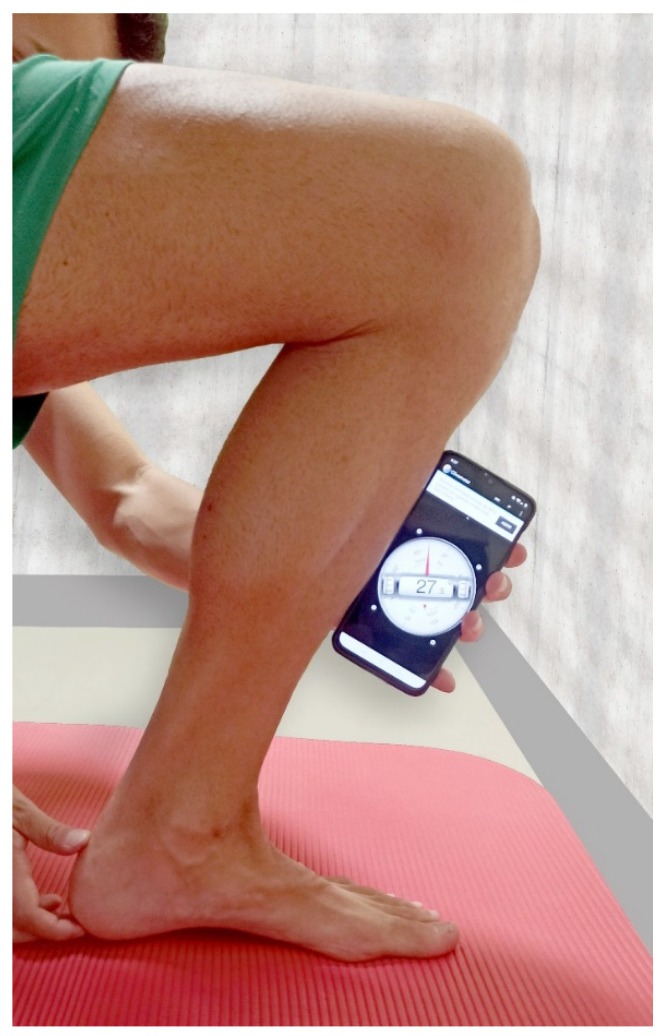
The lunge test was used to measure the ankle dorsiflexion range.

Ankle dorsiflexion range of motion was assessed with the lunge test [9]. The test was carried out by placing the foot perpendicular to a wall, with the sole resting on the ground, and bringing the knee forward towards the wall. The foot was then moved further away from the wall until the maximum range of dorsiflexion was reached. To keep the heel from lifting off the ground, the examiner placed a band under the heel and applied tension (Figure 4), and the angulation from the tibia bone to the ground was measured. This test has shown good intra- and inter-examiner reliability (ICC 0.97) [25].

**Figure 5:**
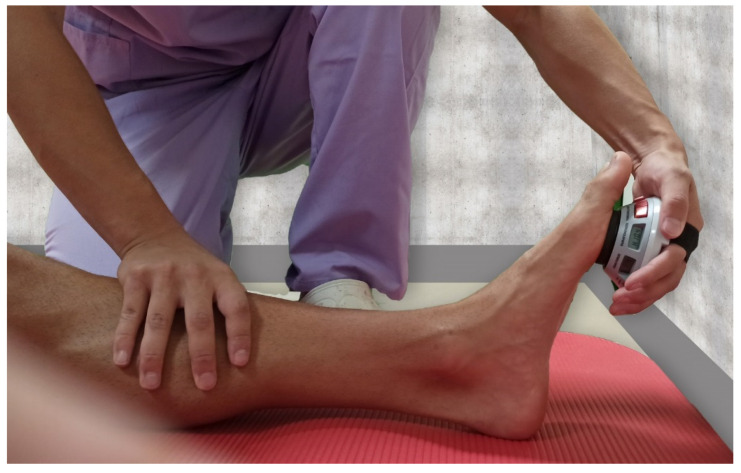
The isometric muscle strength testing with a manual dynamometry.

A dynamometer (MicroFET2, Hoggan Scientific, Salt Lake City, UT, USA) was used for isometric muscle strength testing. All participants were barefoot during the test. The maximum force was measured with the dynamometer, with an accuracy of 0.1 N. The dynamometer was placed over the heads of the metatarsals on the sole of the foot, and the subjects were asked to exert the greatest amount of isometric force possible towards the flexion. Ankle plantar strength was assessed with participants in supine (Figure 5). Each contraction was held for 3 s. The test was repeated 3 times, with a 5 sec-rest, and the mean was calculated [9,26,27].

### 2.5. Dry Needling Intervention

Participants were used as their own controls. One of the lower limbs received the intervention randomly, whereas the other limb acted as a control and did not receive any intervention. Outcomes were evaluated with a 2 min difference between both legs.

**Figure 6:**
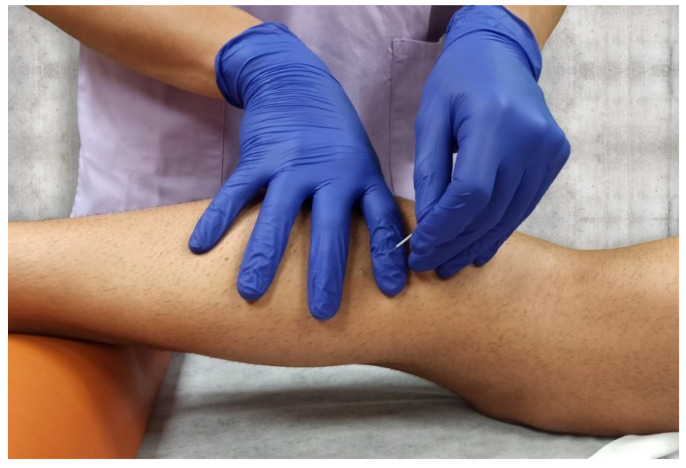
Dry needling procedure on gastrocnemius muscle with “fast-in and fast-out” techniques.

A single dry needling treatment session was performed on the experimental limb in the latent TrP found during the exploration on the gastrocnemius muscle. If multiple TrPs were found, dry needling was applied on the most painful latent TrP in one gastrocnemius muscle. With participants in a prone position, 0.30 mm × 50 mm disposable stainless-steel needles (3B Scientific, Paterna, Spain) were inserted into the skin over the latent TrP (Figure 6).

Once the TrP was located, the skin was cleaned with an antiseptic. In this study, the “fast-in and fast-out” technique, described by Hong [28], was used. The needle was inserted between 5 and 10 mm until the first local twitch response occurred. The technique was performed in different directions for a total of 45 s, and the number of local twitch responses obtained was recorded. Once the needle was removed, manual pressure was immediately applied to the skin area with a cotton ball to generate hemostasis.

### 2.6. Sample Size

The sample size was calculated with the GRANMO version 7.12 software. A previous pilot study (*n* = 10) was conducted to determine the sample size. Subjects used in the pilot study were not included in final trial to avoid potential bias. The primary outcome used for sample size calculation was the muscle tone of the medial gastrocnemius. An initial standard deviation of 0.62 was obtained, a difference between extremities of 0.36 with bilateral contrast, alpha risk of 0.05, beta risk of 0.2, and with a 5% probability of error. The probability of error (1 − β) was 0.80 and the probability of error α was 0.05. With this data, a necessary sample of 50 subjects (100 limbs) was calculated.

### 2.7. Statistical Analysis

Statistical analysis was performed with the SPSS 25.0 package (IBM, Armonk, NY, USA). The Kolmogorov-Smirnov test was used to determine a normal distribution of the quantitative data (*p* > 0.05). A 2 × 2 mixed model of repeated-measures analysis of covariance (ANCOVA) was used to compare the main effect of time (before and after) and group (extremity needled or not needled), with gender as covariate. The main hypothesis of interest was the group × time interaction. A secondary analysis was conducted to determine the effect of gender in the observed changes. Effect sizes were calculated using Cohen’s d coefficient [29]. An effect size of >0.8 was considered large, about 0.5 was considered intermediate, and <0.2 was small [29]. All enrolled subjects were included in the final analysis. The statistical analysis was done by intention to treat (Little’s random complete absence test and maximization of expectations). The level of significance was set at *p* < 0.05.

## 3. Results

**Figure 7:**
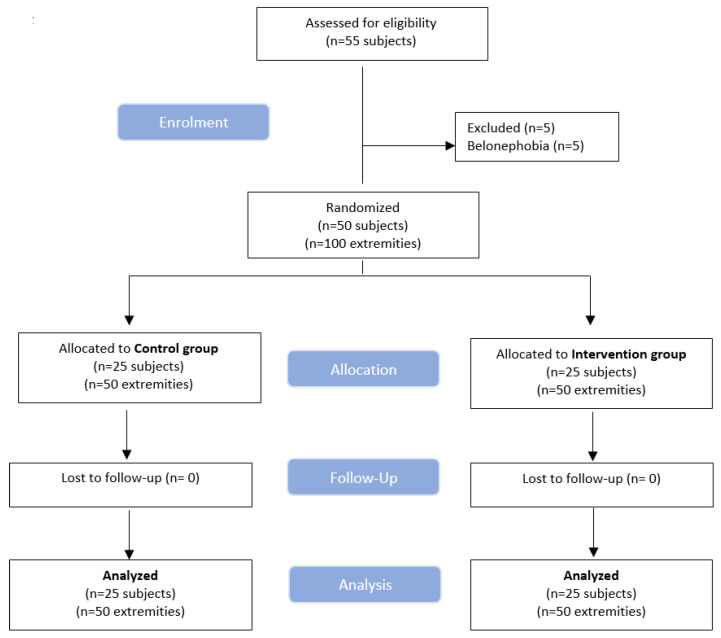
Flow diagram of patients throughout the course of the study, according to the Consolidated Standards of Reporting Trial (CONSORT).

From the 55 consecutive volunteers initially screened, 50 met the inclusion criteria. The details of the recruitment procedure are reflected in the flow chart (Figure 7). The final sample consisted of 15 men and 35 women, mean age (±SD) of 22.4 ± 8.4 years, height 173.1 ± 8 cm, weight 68.9 ± 14.5 kg, and BMI 22.82 ± 3.61 kg/m^2^. The right leg was dominant in 70% of the participants, whereas the left leg was dominant in the remaining 30%. The medial gastrocnemius was needled in 55%, whereas the lateral gastrocnemius was needled in 45% of participants. The mean number of local twitch responses obtained during the needling intervention was 2.5 (SD 3.6) on each TrP. All subjects experienced a transient discomfort after the needling intervention lasting 24–48 h in the targeted area. No other adverse event was reported from any participant.

### 3.1. Changes in Neuromuscular Function—Myotonometry

The ANCOVA revealed significant group × time effects in the lateral gastrocnemius for stiffness (F = 5.928; *p* = 0.02), relaxation (F = 4.113; *p* = 0.045), and creep (F = 5.203; *p* = 0.03), but not for tone (F = 3.353; *p* = 0.073) or elasticity (F = 0.012; *p* = 0.914): the experimental extremity experienced higher decreases in stiffness and higher increases in relaxation and creep in the lateral gastrocnemius, compared to the control extremity (Table 1). No significant effect of time or group was observed for tone (time: F = 0.993, *p* = 0.324; group: F = 0.001, *p* = 0.985) or elasticity (time: F = 0.047, *p* = 0.839; group: F = 0.352, *p* = 0.556) of lateral gastrocnemius. The inclusion of gender did not influence any result (tone: F = 0.423, *p* = 0.529; stiffness: F = 2.416, *p* = 0.127; elasticity: F = 0.003, *p* = 0.954; relaxation: F = 2.755, *p* = 0.105; creep: F = 0.377, *p* = 0.542).

For the medial gastrocnemius, no significant group × time effect, in any outcome, was found (tone: F = 1.079, *p* = 0.304; stiffness: F = 0.176, *p* = 0.677; elasticity: F = 1.071, *p* = 0.306; relaxation: F = 1.179, *p* = 0.283; creep: F = 0.414, *p* = 0.523; Table 1). No significant effect of gender was observed in any outcome of the medial gastrocnemius (all, *p* > 0.209)

### 3.2. Changes in Neuromuscular Function—Tensiomyography

The ANCOVA did not reveal significant group × time interactions for any variable assessed with tensiomyography (TMF), in either lateral (TC: F = 0.163, *p* = 0.689; TD: F = 0.805, *p* = 0.374; TR: F = 0.004, *p* = 0.950; DM: F = 1.142, *p* = 0.290; TS: F = 0.586, *p* = 0.448) or medial (TC: F = 0.428, *p* = 0.516; TD: F = 0.755, *p* = 0.389; TR: F = 0.971, *p* = 0.329; DM: F = 0.269, *p* = 0.606; TS: F = 0.060, *p* = 0.808) gastrocnemius (Table 1). Again, no significant effect of gender was observed (all, *p* > 0.112).

### 3.3. Changes in Sensitivity to Pressure Pain

A significant group × time interaction (F = 5.667; *p* = 0.03) for PPT over the latent TrP was observed: the control extremity experienced a higher increase in PPTs (i.e., decrease in pressure pain sensitivity) than the experimental extremity (Table 2). The inclusion of gender as covariate revealed that women exhibited general lower PPT than men (F = 18.586; *p* < 0.001), but changes after the needling intervention were similar. No significant group × time interaction (F = 0.132; *p* = 0.721), time (F = 2.616; *p* = 0.127), or group (F = 0.132; *p* = 0.721) effect was observed in pain elicited during PPT (Table 2).

### 3.4. Ankle Dorsiflexion (Lunge Test)

The ANCOVA revealed neither a significant group × time interaction (F = 0.930; *p* = 0.340) nor a main effect for time (F = 0.067; *p* = 0.797) or group (F = 0.007; *p* = 0.932, Table 2). The inclusion of gender, as a covariate, did not influence the results (F = 0.235; *p* = 0.630).

### 3.5. Strength

The ANCOVA revealed a significant group × time (F = 6.259; *p* = 0.016), without an effect of gender (F = 0.082; *p* = 0.776): the control extremity experienced a significant decrease in ankle strength when compared with the needle extremity (*p* = 0.003).

## 4. Discussion

The objective of this clinical trial was to evaluate the effects of a single session of dry needling over latent TrPs on neuromuscular function, sensitivity to pressure pain, range of motion, and strength in a sample of asymptomatic subjects. We observed that a single session of dry needling changed some parameters of neuromuscular function, such as muscle tone, relaxation, creep in the lateral (but not medial gastrocnemius), and pressure pain sensitivity, but did not improve strength or range of motion.

### 4.1. Changes in Neuromuscular Function

Our findings observed in myotonometry are similar to those previously observed after the application of manual therapy [9,30] or dry needling [10]. These studies also reported a decrease in muscle tone and an increase of relaxation after the treatment of latent TrPs. Some studies have associated the presence of stiffness with a greater predisposition to experience muscle injuries [31,32,33], which could strengthen the proposed initial hypothesis that treating healthy or subclinical individuals with latent TrPs could be associated with a reduction in the risk of injury [34]. Future studies investigating this hypothesis in sport players are needed, since changes in neuromuscular function were mainly observed in the lateral (but not the medial) gastrocnemius.

We also found a trend towards a decrease of muscle contraction time, as assessed with tensiomyography, particularly in the maximum radial displacement (dm) of the lateral gastrocnemius. Current findings suggest a better muscle efficacy in contractility [35] after the application of dry needling. The decrease in the contraction time at rest, despite not being significant, is a consistent finding observed in previous studies, which deserves future research. Rusu et al. [36] explained that a decrease in contraction time is related to a greater recruitment of muscle fibers. In such a scenario, dry needling could be used as a coadjutant treatment in sport players to induce changes in muscle recruitment. Current results suggest a trend that dry needling could improve muscle contraction and decrease contraction time; nevertheless, these results should be considered preliminary.

Our findings could be interesting for injury prevention. An increase in muscle contraction time has been shown to be related to an increased risk of muscle injuries [37]. Specifically, the speed of contraction of the triceps sural is crucial for stabilizing the knee joint functioning as an agonist in anterior cruciate ligament (ACL) injuries [38,39]. Interventions helping to modify muscle contraction time could help for better or more efficient muscle recruitment during preventive exercise programs in this population.

It is important to consider that we only observed changes in neuromuscular function in the lateral (but not the medial) gastrocnemius. These findings are intriguing. Cadaveric [40] and in vivo [41] studies described differences in the structural characteristics between the medial and lateral gastrocnemius. The medial gastrocnemius has a unipennate structure, whereas the lateral gastrocnemius has a bipennate structure [42]. These anatomical differences could lead to different muscle responses to the application of dry needling. In fact, latent TrPs in the medial (but not lateral) gastrocnemius muscle have been associated with muscle cramps [43]. One hypothesis explaining the pathophysiological effects of latent TrPs on muscle cramps suggests the presence of spontaneous discharges or abnormal excitability from motor nerves and over-excitability of the motor unit in the presence of spinal disinhibition of the medical gastrocnemius muscle [43]. Accordingly, potential differences in excitability of the motor units, between the medial and lateral gastrocnemius, could explain the difference found in our study. It is possible that the medial gastrocnemius needs greater stimulation with the needle, e.g., more local twitch responses or consecutive sessions, for obtaining changes in neuromuscular function. In line with this hypothesis, a preliminary study observed a decrease in superficial electromyographic activity of the medial gastrocnemius with consecutive local twitch responses elicited during the application of dry needling over latent TrPs [44]. Future studies investigating the effects of consecutive treatment sessions or higher number of local twitch responses would help to elucidate this hypothesis.

### 4.2. Changes in Sensory and Motor Outcomes

We observed a small increase in PPT over the latent TrPs and a decrease on the pain intensity perceived (NPRS) after a single session of dry needling, although these changes did not reach neither statistical significance nor clinical relevance. These small changes could likely be associated with type I errors.

Interestingly, the control leg experiment showed a higher increase in PPT, probably related to post-needling pain soreness associated with the intervention, due to the described tissue damage [45]. Our results would agree with the fact that manual therapies are more effective for immediate effects on pain sensitivity [9,34]. This contralateral increase in PPT observed may be due to the potential spinal neurophysiological effect of dry needling [46]. It has been reported that manual techniques generate hypoalgesia, changes in electrical conduction, and changes in muscle properties both locally and distally to the treated area [47,48,49,50,51]. These mechanisms occur through segmental inhibitory pathways, spinal pathways, or descending inhibitory pathways [52,53]. These changes would explain the presence of contralateral changes in sensitivity to pressure pain in the current study. However, despite these findings, it seems clear that changes in the treated areas seem to be more substantial than those in distant areas [54,55,56].

No significant changes in range of motion were found, either. A single treatment session may not be enough to obtain the range of motion changes which have been reported by others [57]. In fact, differences are found when a greater number of sessions are applied [58]. Another possible reason may be that the patients did not exhibit a reduction in range of motion prior to the needling intervention, as they were asymptomatic. Therefore, it is possible that changes in range of motion would occur if dry needling were applied on symptomatic populations. Finally, it should be also considered that the small changes seen after the intervention could be likely represent type I errors.

Finally, regarding strength, we observed a decrease of ankle strength within the control group. These data are similar to a previous study [9] and to a previous meta-analysis showing that most of the studies suggest no effect of dry needling on force production [59]. It is possible that the between-groups differences are due to the fact that the TrPs have not been treated in the control group and the evaluation itself has generated the irritation of muscle tissue, leading to a decrease in strength; however, these data are inconclusive. Again, the possibility of type I error is present.

### 4.3. Limitations

We should consider that this study included people with latent TrPs; therefore, they did not exhibit any painful pathology. This sample does not represent usual clinical practice. It would be interesting to conduct future studies, including pain populations exhibiting active TrPs. Second, we only evaluated the immediate effects of the intervention, so we do not know if the results will be maintained in subsequent follow-ups or if they will disappear. Furthermore, only the effect of a single treatment session was assessed, so it is possible that carrying out multiple sessions may lead to better results. Finally, it is probable that type I errors could be present, mostly in secondary outcomes.

## 5. Conclusions

The results of this study indicate that a single dry needling session on latent TrPs can improve some parameters of neuromuscular function, such as tone, relaxation, creep, and pressure pain, but not improve strength or range of motion. These changes were identified for the lateral, but not the medial, gastrocnemius.

## Figures and Tables

**Table 1:** Mean changes in neuromuscular function, according to myotonometry and tensiomyography.

	Intervention Extremity (Group)					Control Extremity (Group)				
	Baseline	Final	Within-Group Difference (Pre-Post)			Baseline	Final	Within-Group Difference (Pre-Post)		
	Mean ± SD	Mean ± SD	Mean	95% CI	ES	Mean ± SD	Mean ± SD	Mean	95% CI	ES
**Lateral Gastrocnemius**										
Tone (Hz)	15.88 ± 2.08	15.48 ± 1.95	−0.36	(−0.545; −0.173)	0.20	15.67 ± 2.72	15.69 ± 1.97	0.06	(−0.440; 0.564)	0.01
Stiffness (N/m) *	283.72 ± 47.64	274.10 ± 43.58	−8.28	(−12.838; −3.725)	0.21	279.72 ± 45.29	276.02 ± 43.09	−2.65	(−7.423; 2.125)	0.08
Elasticity	1.17 ± 0.19	1.19 ± 0.18	0.02	(−0.002; 0.059)	0.11	1.16 ± 0.20	1.18 ± 0.19	0.02	(−0.002; 0.055)	0.10
Relaxation (m/s) *	19.56 ± 3.56	20.13 ± 3.13	0.50	(0.136; 0.863)	0.17	19.80 ± 3.45	19.97 ± 3.11	0.11	(−0.252; 0.471)	0.05
Creep (m/s) *	1.20 ± 0.21	1.23 ± 0.18	0.03	(0.006; 0.052)	0.15	1.23 ± 0.25	1.22 ± 0.18	−0.02	(−0.063; 0.029)	0.05
TC (m/s)	35.59 ± 20.72	37.57 ± 21.38	1.98	(−2.958; 6.913)	0.09	34.62 ± 21.69	38.04 ± 21.60	3.43	(−2.431; 9.285)	0.16
TD (m/s)	23.54 ± 18.68	21.69 ± 3.83	−1.85	(−7.322; 3.629)	0.14	20.75 ± 3.14	22.18 ± 7.22	1.43	(−0.519; 3.381)	0.26
TR (m/s)	59.70 ± 36.97	54.51 ± 42.05	−5.19	(−20.199; 9.810)	0.13	52.78 ± 34.68	48.18 ± 22.34	−4.60	(−14.777; 5.571)	0.16
DM (mm)	3.46 ± 2.25	3.47 ± 2.48	0.01	(−0.374; 0.401)	0.00	3.41 ± 2.22	3.71 ± 2.38	0.30	(−0.165; 0.772)	0.13
TS (m/s)	231.21 ± 56.24	222.33 ± 50.75	−8.88	(−30.636; 12.868)	0.17	226.48 ± 68.99	208.50 ± 6.74	−17.98	(−39.849; 3.879)	0.37
**Medial Gastrocnemius**										
Tone (Hz)	15.09 ± 1.66	14.70 ± 1.61	−0.38	(−0.554; −0.208)	0.24	15.17 ± 1.55	14.87 ± 1.57	−0.28	(−0.438; −0.113)	0.001
Stiffness (N/m)	254.48 ± 54.46	250.52 ± 34.47	−3.42	(−14.70; 7.856)	0.09	258.24 ± 36.35	252.38 ± 33.58	−5.26	(−10.101; −0.421)	0.034
Elasticity	1.17 ± 0.18	1.18 ± 0.14	0.01	(−0.039; 0.064)	0.06	1.13 ± 0.22	1.18 ± 0.14	0.05	(−0.003; 0.098)	0.064
Relaxation (m/s)	20.88 ± 3.13	21.51 ± 2.79	0.59	(0.220; 0.955)	0.13	20.86 ± 3.07	21.35 ± 2.77	0.43	(0.067; 0.798)	0.021
Creep (m/s)	1.27 ± 0.18	1.30 ± 0.16	0.03	(0.009; 0.054)	0.18	1.26 ± 0.18	1.29 ± 0.16	0.03	(0.002; 0.049)	0.032
TC (m/s)	22.68 ± 10.62	22.60 ± 7.71	−0.08	(−1.953; 1.797)	0.01	24.14 ± 12.60	23.34 ± 10.30	−1.02	(−3.276; 1.329)	0.369
TD (m/s)	19.33 ± 2.20	19.21 ± 2.41	−0.11	(−0.640; 0.417)	0.05	20.45 ± 8.05	19.29 ± 2.99	−1.24	(−3.769; 1.293)	0.330
TR (m/s)	59.38 ± 70.41	58.05 ± 55.33	−1.33	(−22.370; 19.705)	0.02	75.30 ± 137.71	52.59 ± 58.37	−24.12	(−62.792; 14.552)	0.216
DM (mm)	1.87 ± 1.25	1.72 ± 1.24	−0.16	(−0.309; −0.007)	0.12	1.87 ± 1.24	1.81 ± 1.17	−0.09	(−0.317; 0.132)	0.412
TS (m/s)	203.80 ± 96.94	211.15 ± 92.63	7.35	(−12.566; 27.256)	0.08	212.72 ± 170.68	227.31 ± 104.74	10.69	(−44.470; 65.450)	0.696

DM: maximum radial displacement; TC: contraction time; TD: delay time; TR: relaxation time; TS: sustained time; ES. Effect Size. * Significant group × time interaction (ANCOVA, *p* < 0.05).

**Table 2:** Mean changes of the lunge test, strength, and pressure pain sensitivity in both extremities.

	Intervention					Control				
	Baseline	Final	Difference between Baseline			Baseline	Final	Difference between Baseline		
	Mean ± SD	Mean ± SD	Mean	95% CI	ES	Mean ± SD	Mean ± SD	Mean	95% CI	ES
LUNGE (°)	40.86 ± 6.18	41.32 ± 7.05	0.46	(−0.557; 1.481)	0.07	41.15 ± 6.79	40.94 ± 7.45	−0.22	(−1.552; 1.120)	0.03
FORCE (N) *	43.57 ± 10.94	43.05 ± 11.92	−0.68	(−2.875; 0.151)	0.05	44.94 ± 11.55	41.68 ± 12.19	−3.26	(−5.352; −1.177)	0.28
PPT (Kg/cm^2^) *	5.35 ± 2.56	5.57 ± 2.84	0.22	(−0.226; 0.660)	0.08	5.33 ± 2.50	6.45 ± 2.63	1.13	(0.374; 1.876)	0.44
NPRS (0–10)	4.12 ± 1.87	3.14 ± 1.38	−0.97	(−1.468; −0.479)	0.60	3.28 ± 1.13	2.75 ± 0.97	−0.53	(−1.162; 0.112)	0.50

PPT: Pressure Pain Threshold; NPRS: Numerical Pain Rating Scale; N: Newton; ES: Effect Size. * Significant group × time interaction (ANCOVA, *p* < 0.05).
